# Inequalities in neighbourhood features within children’s 20-minute neighbourhoods and variation in time spent locally, measured using GPS

**DOI:** 10.1016/j.wss.2023.100174

**Published:** 2023-12

**Authors:** Jonathan R Olsen, Fiona Caryl, Natalie Nicholls, Melody Smith, Paul McCrorie, Richard Mitchell

**Affiliations:** a MRC/CSO Social and Public Health Sciences, University of Glasgow, Glasgow, UK; b Faculty of Medical and Health Sciences, University of Auckland, Auckland, New Zealand

**Keywords:** inequalities, children, 20-minute neighbourhood, urban, rural, urban design

## Abstract

There has been a growing interest in policies that encourage local living by promoting accessible and walkable communities, such as the 20-minute neighbourhood concept. Despite the widespread adoption of this policy in cities worldwide, little research has been conducted on the characteristics of children’s 20-minute neighbourhoods and their association with time spent locally.

This study aimed to explore the features of Scottish children’s 20-minute neighbourhoods by analysing an 800-meter road and path network buffer surrounding 687 children’s homes. Based on existing literature, the study identified key features associated with children’s time spent locally and the 20-minute neighbourhood policy. The study then examined variations in these features by socioeconomic status, urbanicity, and gender.

The findings revealed significant inequalities in the presence of health-benefiting (e.g., green spaces, recreational facilities, healthy food outlets) and health-harming (e.g., major roads, unhealthy commodity retailers) environments within children’s 20-minute neighbourhoods. Children from more deprived areas had access to more of both types of environments. The study also found that having a school within a 20-minute neighbourhood was associated with an increased amount of time spent locally (IRR 1.62, 95% CI 1.5 to 1.8, p<0.001).

The study suggests that the 20-minute neighbourhood policy should extend beyond mere access to local amenities and prioritise creating healthy 20-minute neighbourhoods, particularly in socioeconomically deprived areas. The research highlights the importance of promoting equal access to quality local environments, which can contribute to improved health and well-being outcomes for children.

## 1 Introduction

There has been a recent and renewed interest in planning policies that focus on local living, specifically concepts such as *20-minute neighbourhoods* or the *‘X’-minute city*. By the end of 2020, over 33 global cities had implemented or were considering adopting the 20-minute neighbourhood policy into their development plans (Gower and Grodach 2022). The policy is rooted in a compact city development that encourages places to be designed to provide communities and their residents access to well-connected facilities and amenities, such as education, essential services, shopping, open spaces and public transport, within a short walk to their homes to enable daily local living (Chau *et al.* 2022; Giles-Corti *et al.* 2016). A 20-minute neighbourhood policy means these facilities and amenities can be accessed within a 10-minute walk to-and-from a residential location (Thornton *et al.* 2022). An 800m distance is a common metric that generally aligns to a 10-minute walk based on average walking speeds and, for children, is considered a reasonable buffer distance to characterise a directly accessible environment (Dessing *et al.* 2016; Finnis and Walton 2008; Yang and Diez-Roux 2012). The ‘X’-minute city and 20-minute neighbourhood are based on the same planning concepts, the differences in terminology stem from the walking time threshold (minutes) and scale of implementation (city, state or nationally) planning authorities target in providing access to local amenities for their residents (Logan *et al.* 2022).

There are a number of suggested benefits to planning policies focusing on local living. These include decreasing health inequalities, as well as improving the local economy, improving liveable quality of life and reducing the impact of climate change through less dependency on car travel and increases in walking and wheeling (O’ Gorman and Dillon-Robinson 2021). For children, evidence has been increasing that having access to specific facilities and amenities is important for spending greater time in or visiting an area. For example, children have been found to spend a greater amount of time in locations that contained schools, public transit stops, food/drink retail, places of worship, libraries, recreational facilities, natural spaces and parks (Chambers *et al.* 2017; Egli *et al.* 2020; Loebach and Gilliland 2016; McCrorie *et al.* 2021; Villanueva *et al.* 2013). However, it is important that access to potentially health benefiting facilities is not viewed in isolation; evidence from New Zealand has shown that areas with a greater number of health benefiting facilities and amenities also often co-contain health harming facilities (Marek *et al.* 2021).

In a formal assessment of spatial access to ten domains of services and amenities for all residential locations in Scotland, a socioeconomic gradient was observed which ran in the opposite direction that we may have expected. Across all ten domains, a higher proportion of residential locations in the most deprived areas had greater access to the services and amenities within their 20-minute neighbourhood than those in the least deprived areas (J.R. Olsen *et al.* 2022). However, this relationship may not be straight-forward and positive. For example, children from lower income households had greater exposure to unhealthy commodity advertising at transport stop locations, which clustered in urban residential areas (Liu *et al.* 2022). Further, not all retailing is healthy retailing. Children from the most deprived areas experience significantly more exposure to shops selling tobacco and alcohol products than children from the least deprived areas (Caryl *et al.* 2019).

The home neighbourhood is an important place where children spend their time; in New Zealand, children were found to spend 50% of their time within a 500m buffer of their home (Chambers *et al.* 2017). However, there is little evidence relating to whether children who have access to key facilities and amenities within a walkable 800m distance from their homes use them or spend more time in their local area. The limited evidence base suggests a deeper understanding of how local populations use and view existing infrastructure is required to support local living and 20-minute neighbourhood policies. By way of example, a study examining access to, and use of, public parks found distance to the closest park was not associated with park use (Kaczynski *et al.* 2014).

Despite the adoption of the 20-minute neighbourhood policy in many cities globally, there is surprisingly little evidence describing the contents of 20-minute neighbourhoods and variation by sociodemographic factors. As well as whether having access to facilities and amenities within a 20-minute neighbourhood is associated with increased time spent living locally, or alternatively, whether specific features may increase or decrease time in those neighbourhoods. The aim of this study is to contribute to filling these important knowledge gaps.

### Study objectives

(1) Create 800m road and path home network buffers for children in Scotland to define their 20-minute neighbourhood.
(2) Identify a comprehensive list of spatial features associated with children’s time spent living locally.
(3) Describe variation in spatial features within children’s 20-minute neighbourhoods by sex, socio-economic and urban/rural status.
(4) Link detailed mobility data for children to their home network buffers to describe the proportion of time spent within their 20-minute neighbourhood by weekday, weekend and overall.
(5) Explore whether specific spatial features within children home network buffers are associated with more or less time spent within their 20-minute neighbourhood.

## 2 Methods

### 2.1 Study setting and participants

The study used data from the SPACES (Studying Physical Activity in Children’s Environments across Scotland) study (University of Glasgow 2023). SPACES is a national cross-sectional dataset in Scotland, a country and devolved administration within the UK. Scotland has a population of 5.5 million (16 years and under: 968,802 (17.7%)) and covers an area of 77,911 km2 (National Records of Scotland 2022). The SPACES dataset provided detailed mobility data for Scottish children linked to geocoded home and school address locations. Briefly, SPACES sub-sampled participants from Growing up in Scotland (GUS) Birth Cohort 1 (BC1); an on-going Scottish cohort study that began in 2004 (McCrorie *et al.* 2018). The original GUS sample (n=5217) method ensured national representativeness across socioeconomic situation. For this study, participants’ characteristics, such as age, sex and household income were provided by the GUS dataset. A total of 687 children were included within this study and formal data collected was scheduled during school term times.

### 2.2 Defining children’s 20-minute neighbourhoods

Network buffers were created surrounding the individual home location of all SPACES children within the Network Analyst extension (ArcGIS Pro 2.9.2) using the road and path network (Integrated Transport Network (ITN) Layer, OS MasterMap). One-way and turn restrictions for motorised traffic were removed to better model pedestrian travel. An 800-meter network distance was used as this is the boundary specified within the Scottish Government’s (draft) Fourth National Planning Framework (Scottish Government 2020a) and a commonly applied globally when referring to a 20-minute neighbourhood (Thornton *et al.* 2022), which suggests a 10-minute walk to-and-from a destination. Having delineated a 20-minute neighbourhood for each child, we then quantified characteristics within those neighbourhoods (described below).

We also created an 800m Euclidean buffer around each child’s home to conduct a sensitivity analysis of the features within both a walkable home network buffer (that may be a different geographical area for each child based on the road/path density) and a standard ‘as the crow flies’ circular buffer (that will be the same geographical area for each child but may not be within a 10-minute walk).

### 2.3 Neighbourhood characteristics

We identified neighbourhood characteristics associated with children’s time spent locally (both increased and decreased) from available literature and the Healthy Environments Index for Children (Whitehead *et al.* 2023), along with those highlighted within 20-minute neighbourhood policies (Gower and Grodach 2022; Thornton *et al.* 2022) (Table 1). To quantify each feature (Table 1) inputs were spatially joined to each child’s 20-minute neighbourhood boundary. We also calculated and report the size (meters squared (m^2^)) of each child’s 20-minute neighbourhood.

A measure of neighbourhood diversity, based on the Shannon Index, was created from the following non-residential land features: manufacturing and production, public infrastructure (including education and health), non-food and retail commercial services, retail (food and non-retail commercial services) and open space (‘bodies of water’; landscape features; recreational features.). This measure was calculated at child level – that is, from the counts of the above features within 800m of the child’s home location, based on the equation:

$$-\sum_{i=1}^{N}p_{i}\ln\left(p_{i}\right)$$

Where N is the number of non-residential features (here 5), p is the proportion of feature type i, and is calculated by dividing the number of features of type i by the total number of features. The higher the value, the more diverse the non-residential environment around the child’s home location.

Area-level socioeconomic situation and urbanicity was joined to each child’s home location using the income domain of the Scottish Index of Multiple Deprivation (SIMD) (Scottish Government 2020b). The Scottish Government’s six-fold urban/rural classification was used to specific urbanicity (Scottish Government 2018).

The shortest home-to-school network distance (km) was calculated from each child’s home location to their school using the *gmapsdistance package55* within R V.3.2.0. Using this output, we computed whether the child’s school was within 800m of their home.

### 2.4 Location measurement using Global Positioning System (GPS) device

In the SPACES study, from which our data were drawn, children were provided with a GPS device (Qstarz BT-Q1000XT; Qstarz International Co., Ltd, Taiwan) and asked to wear the device over eight consecutive days during waking hours. The GPS devices have a median location error of 2.5m and are found to be acceptable for use in larger population studies, especially with relatively long data collection periods (7 days or more) (Schipperijn *et al.* 2014). The device recorded the child’s point location every 10 seconds. We refer to each recorded location as a ‘point’.

### 2.5 Spatial data linkage

The GPS points for each child (~16 million points in total, median: 24258) were spatially joined to their home-based 800m/20-minute neighbourhood network boundary using the sf R package (Pebesma 2018). GPS points were classified into binary (yes/no) attributes according to whether the GPS point was either; (i) ≤800m of their home, or (ii) >800m from the home buffer. The date of each GPS point was used to allow us to group by weekday (Monday to Friday), weekend (Saturday and Sunday) and overall (Monday to Sunday).

### 2.6 Descriptive analysis

Participants’ characteristics are described (sex, area-level socioeconomic status and urban/rural classification of home datazone) as well as the proportion of total GPS wear time spent within 800m of home by weekday, weekend and overall. Summary measures were calculated overall and by sex, socioeconomic status and urban/rural classification for all the neighbourhood characteristics (Table 1). We explored differences in the characteristics within each child’s 20-minute neighbourhood by sex, socioeconomic status and urban/rural classification.

### 2.7 Statistical analysis

To formally test whether neighbourhood characteristics were associated with increased/decreased time spent locally, time spent within 800m of home, overall, on weekdays and on weekends, were set as the outcomes in separate complex sample negative binomial models – accounting for the clustered and stratified survey sample design of the GUS cohort. The log of total wear time was set as the offset, to account for differences in device wear between the children. The main independent variables of interest were the neighbourhood features defined in Table 1 and results are presented as incident rate ratios (IRR) In addition, all models were controlled for sex, whether the school attended was within 800m of home, area-level socioeconomic status, urban/rural location, the non-residential diversity index (described above) and sample weighting was applied. The diversity measure was used as a proxy for the combination of all non-residential features present. All analyses were performed in Stata 17, using the svy syntax, with a global significance level set at 5%.

## 3 Results

### 3.1 Neighbourhood characteristics of children’s 20-minute neighbourhoods by sex, socioeconomic status and urbanicity

#### 3.1.1 Size of 20-minute neighbourhood

The size of children’s 20-minute neighbourhoods (in terms of area (m^2^)) varied by socio-economic status and urbanicity (Table 2). Children living the most deprived areas had 25% larger 20-minute neighbourhoods (1,188,297m^2^) than those living in the least deprived areas (886,110m^2^). Similarly, children living in large urban areas (1,106,920m^2^) had 50% larger 20-minute neighbourhoods than those living in remote rural areas (556,161m^2^). Highlighting that urban areas, which typically have a greater road and path density, create 20-minute neighbourhoods that have a larger geographical area accessible within a 10-minute walk (800m).

#### 3.1.2 School within 20-minute neighbourhood

Overall, 34% of children lived 800m or less from the school they attended (Table 2). However, a greater proportion (49%) of those residing in the most deprived areas lived 800m or less to their school, compared to a quarter (23%) of those living in the least deprived areas. Surprisingly, a greater proportion of children from remote rural areas of Scotland lived within 800m of their primary school (70%) than in large urban areas (33%); although remote rural areas represented a smaller number of participants. In terms of having access to a primary school (regardless of attending that school), children from the most deprived areas had more schools (median=2) within their 20-minute neighbourhood compared to children living in the least deprived areas (median=0).

#### 3.1.3 Urban density and transport

Children from the most deprived areas lived in areas of greater residential density, with almost double the number of residential dwellings (median count: 2,169) compared to the least deprived areas (median count: 1,023) and had almost three times as many public transport stops (median count: most deprived: 28; least deprived: 10). Children living in the most deprived areas had over double the length (meters) of major roads within their 20-minute neighbourhood (median meters: most deprived: 1,272; least deprived: 512) and 48% more minor road meters (median meters: most deprived: 14,804; least deprived: 9,483).

#### 3.1.4 Retail

The median count of all retail outlets within Scottish children’s 20-minute neighbourhoods was 4. For non-food, healthy food, and unhealthy food & drink retail there was a greater median count for children living in the most deprived areas. On average, children living in the least deprived areas had zero healthy food retailers within their 20-minute neighbourhood compared to 1 for children in the most deprived areas. However, for unhealthy food and drink retailers, the median count was 4 times higher for children in the most deprived area (n:4) compared to the least deprived areas (n:1).

A similar relationship was apparent for amenities, greenspace and recreation facilities where children living in the most deprived areas had better access compared to the least deprived areas (Table 2). Although libraries have a median of 0 overall, there were cases of some children having a library within their 20-minute neighbourhood.

#### 3.1.5 Comparison of 20-minute neighbourhood characteristics with 800m Euclidean buffer

Supplementary Table 1 presents the neighbourhood characteristics using 800m Euclidean buffers around children’s homes. There are a greater number of neighbourhood features within the Euclidean buffer compared to network buffer. These may not be accessible within a 10-minute walk of the child’s home as they do not use the road and path network, instead a circular buffer around the home. There is a similar pattern in a greater amount of neighbourhood features by sex, socioeconomic and urban/rural status across the two buffers.

### 3.2 GPS wear time within 20-minute neighbourhoods by weekday/weekend, sex, socioeconomic status and urbanicity

**Figure 1:**
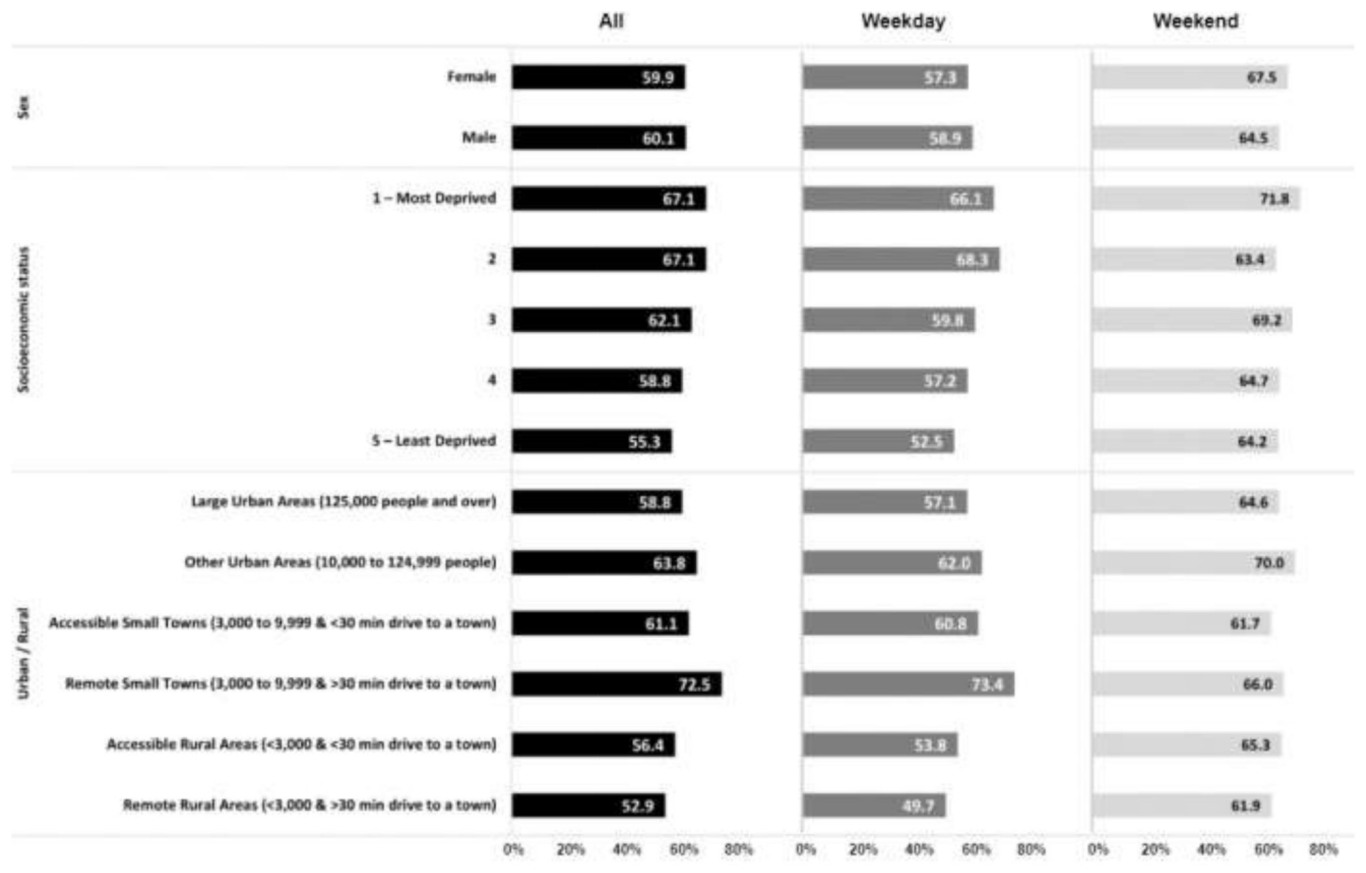
Proportion of time spent within 20-minute neighbourhoods by sex, socioeconomic status and urbanicity.

Overall, 60% of all children’s GPS wear time was within their 20-minute neighbourhood (Figure 1; Supplementary Table 2). Children spend more of their time within their 20-minute neighbourhood on weekdays (66%) compared to weekend days (58%). There was little variation by sex across the measurement periods. However, there was variation by socioeconomic status across all time periods; children living in the most deprived areas spent a greater proportion of their wear time within their 20-minute neighbourhood compared to those resident in the least deprived areas (weekday: Most deprived: 66%, Least Deprived 53%). There was a smaller variation by urbanicity, than by deprivation (weekday: Large Urban Areas 57%, Remote Rural Areas 50%).

### 3.3 Association between presence of neighbourhood characteristics within 800m of home and GPS wear time within 800m of home

Table 3 presents the results of a model including sex, area-level socioeconomic situation, urbanicity, non-residential diversity and school (attends) location to explore whether these factors are associated with the proportion of wear time spent within a 20-minute neighbourhood.

Attending a school within 800m of home had the strongest association with spending time within a 20-minute neighbourhood, overall (IRR 1.62, 95% CI 1.5 to 1.8, p<0.001) and on weekdays (IRR 1.87, 95% CI 1.7 to 2.1, p<0.001), though the association was not as strong at weekends, when children would not be attending school. No association was found with sex, or the non-residential diversity index and time spent within a 20-minute neighbourhood. Children from rural locations were less likely to spend time within their 20-minute neighbourhood than those living in urban areas overall (IRR 0.92, 95% CI 0.9 to 0.98, p=0.02) and on weekdays (IRR 0.9, 95% CI 0.8 to 0.98, p=0.02). Children from more deprived areas were more likely to spend time nearer their homes than those from less deprived areas (Overall: IRR 1.14, 95% CI 1.0 to 1.3, p=0.003).

Finally, individual neighbourhood characteristics were modelled both individual and together, whilst adjusting for multiple tests of various features. No associations with any of the neighbourhood features and increased wear time within 20-minute neighbourhoods were detected, except for the school location (Supplementary Table 3).

## 4 Discussion

The aims of this study were to characterise the features within Scottish children’s 20-minute neighbourhoods, based on an 800m road and path network buffer surrounding 687 children’s home locations, and examine variations in both features and time spent by sex, socio-economic situation and urbanicity.

Children living in the most deprived areas tended to have a greater number of facilities and amenities within their 20-minute neighbourhood compared to those living in the least deprived areas. This included both health benefiting facilities, such as health care providers, green spaces, and healthy food retailers, as well as health harming environments, including a greater sum of minor/major roads and unhealthy food and drink retailers.

Overall, children spent 60% of their total GPS wear time within their 20-minute neighbourhood. This was greater during weekdays (66%) compared to weekends (58%), probably driven by attending school during the week. Children from the most deprived areas spent more of their wear time overall within their 20-minute neighbourhood (Most deprived: 67%; Least deprived: 55%), on weekdays (Most deprived: 66%; Least deprived: 53%) and during weekdays (Most deprived: 72%; Least deprived: 64%) compared to the least deprived. There was little variation by sex.

Children who attended a school within their 20-minute neighbourhood inevitably spent a greater proportion of their GPS wear time within this area, this was also the only neighbourhood feature associated with increased GPS wear time spent locally.

### 4.1 Comparison with other literature

We found that 34% of children attended school within 800m of their home. This was higher for children living in the most deprived areas (49%) compared to the least deprived areas (23%). The proximity of a child’s school to their home has been consistently associated with increased active travel (walking or wheeling) to school (Wangzom *et al.* 2023). Decreasing car dependency and increasing journeys actively travelled is a key outcome of a 20-minute neighbourhood. Studies in Canada and Scotland have shown that the proportion of children who actively travelled to school was between 73-84% when the home-to-school distance was less than 800m (Guliani *et al.* 2015; Waygood and Susilo 2015). In New Zealand 35% of adolescents (mean age 15 years) home-to-school distance was less than 2.25km and 64% of those actively travelled. This decreased to 18% where the home-to-school distance increased from 2.25 to 4km (Mandic *et al.* 2023). In addition to home-to-school distances, other built environmental factors are important to promote children’s active travel to school, such as convenient, safe and connected walking and cycling infrastructure (Winters *et al.* 2017).

We found that urban, densely populated areas created larger 20-minute neighbourhoods, meaning that the geographical area that could be reached within a 10-minute walk (800m road and path network buffer) varied by both urban/rural and socioeconomic status. As 88.7% of the most deprived areas within Scotland are located in large or other urban areas (Scottish Goverment 2020), it is inevitable they would create larger, walkable areas. Studies have highlighted that residential areas with high walkability scores are usually located in urban areas that have greater access to amenities (Xia *et al.* 2018).

Our study found the 20-minute neighbourhoods of children living in the most deprived areas of Scotland offered a greater availability of facilities; increased number of green spaces and a larger sum of roads, compared to those living in the least deprived areas. These factors have been associated with children’s physical activity, active travel and BMI. Ortegon-Sanchez *et al.* (2021) in their review of the built environment and children’s health found that greater availability of greenspaces and recreational resources were associated with increased physical activity. On the other hand, higher traffic levels and increased food outlets/retail density were associated with decreased physical activity, active travel, and BMI. The review also noted that factors such as perceived personal safety, social support, walking infrastructure, traffic calming and traffic levels are important considerations for children’s health behaviour and outcomes (Ortegon-Sanchez *et al.* 2021). Lack of safety from traffic is a key deterrent to active school travel (Carroll *et al.* 2015) and feeling safe in the neighbourhood permeates children’s perceptions and experiences as well as a key component of healthy neighbourhoods (Williams *et al.* 2022). Environmental injustices have been highlighted in relation to air pollution from car traffic where polluting emissions were found to be highest in the areas containing the most income deprived households across England and Wales, whom also had the lowest rates of car ownership (Barnes *et al.* 2019). These studies highlight the importance of moving beyond considering accessibility to destinations alone (as we have done here) and that other place-making factors are key to creating healthy 20-minute neighbourhoods for children.

Our findings add to the growing literature about colocation of both healthy and unhealthy amenities / facilities. Research from the UK, for example, has highlighted the clustering of unhealthy retailers selling alcohol, tobacco, fast food and gambling outlets in deprived areas (Macdonald *et al.* 2018), whilst further evidence from New Zealand suggests that health-promoting and health-constraining environments often collocate (Marek *et al.* 2021). We highlight that there is a greater density of unhealthy commodities within the home neighbourhoods of children from deprived areas, which are also the areas where children spend the majority of their time.

There was no meaningful difference in the proportion of time spent in the 20-minute neighbourhood by sex. This was somewhat unanticipated considering social constructions of gender and associated socio-cultural norms about children’s mobility (Murray 2009), which could be expected to lead to reduced mobility for girls (Marzi *et al.* 2018). Inconsistent findings have been reported regarding mobility equity by sex or gender in children – the landmark Policy Studies Institute study reported no significant sex differences in independent mobility (Shaw *et al.* 2015). Conversely, Marzi *et al.* (2018)’s systematic review determined that sex and gender were important predictors of children’s independent mobility. Differential relationships have been observed by sex in terms of the social and physical environments that impact mobility (Foster *et al.* 2014; Medeiros *et al.* 2021), and in the types of mobility undertaken (Goel *et al.* 2023). In this study we found no significant difference by sex in proportion of time spent in the 20-minute neighbourhood based on the availability of a range of different environmental characteristics. These findings align with previous reviews and empirical studies indicating that while some differences exist, overall, younger children (such as in this study) appear to have less pronounced sex or gender differences in mobility, and that gender disparity in mobility increases with age (Goel *et al.* 2023). It is noteworthy that we only focused on time spent in the neighbourhood, and we did not examine differences by key mobility measures such as independent mobility or active transport – it is possible that time spent in the neighbourhood was a combination of motorised and active mobility. Finally, gender was not assessed in this study. Future research would benefit from considering gender mobility equity and associated policy implications in a targeted manner, particularly in studies that include older children than those in this study.

### 4.2 Policy impact

Our findings support some aspects of the 20-minute neighbourhood idea, highlighting that if a child’s school is within their 20-minute neighbourhood, they will spend more time within their local area. Residential proximity to school has also been shown to increase the likelihood of children actively travelling there. We found that the size of a 20-minute neighbourhood varies, with children living in the most deprived and urban areas having a greater geographical area they can reach within a 10-minute walk. However, our findings also provide important considerations for implementation of the 20-minute neighbourhood policy. We draw attention to the colocation of facilities and amenities that may benefit local living with harmful features, such as busy roads and unhealthy food/drink retailers. This suggests that 20-minute neighbourhood policies must look beyond simple presence or absence of features to consider that alternative policy approaches may be required if 20-minute neighbourhoods are going to be ***healthy.*** Policy must consider wider aspects of place-making in tandem with a 20-minute focus. These may include: limiting the density of health harming facilities (fast-food, alcohol, tobacco outlets), particularly in more deprived areas where they tend to cluster (Macdonald *et al.* 2018; Marek *et al.* 2021); implementation of low traffic neighbourhoods that restrict through motor traffic from residential streets within a neighbourhood (Aldred *et al.* 2021) (which have been shown to both increase walking/cycling and decrease road traffic accidents (Laverty *et al.* 2021)); and 20mph/30kmh speed restrictions in residential areas that are effective in reducing the number and severity of collisions and casualties (Cleland *et al.* 2020). Other place-making qualities should be considered, such as care and maintenance of local areas, sense of community identity, belonging, social contact and the quality of local infrastructure (Hasler 2018).

### 4.3 Strengths and limitations

Our study had several strengths. Our study quantified individual-level 20-minute neighbourhoods with a focus on environments that might matter most for children, and combine this with detailed mobility data from GPS devices to provide a direct policy evaluation of the 20-minute neighbourhood concept. We considered many neighbourhood factors, identified from the literature, that have been associated with children spending more or less time within their home neighbourhood. We used road and path network buffers to provide an estimate of the walkable 800m area surrounding a home. We also conducted a sensitivity analysis by comparing these to a standard 800m Euclidean buffer and found a similar pattern in our results. Our study included a subset of a national cohort, covering both urban and rural areas of Scotland. By including GPS data, we were able to describe the time spent within each child’s 20-minute neighbourhood and understand whether specific neighbourhood features were associated were more or less time spent there. Finally, we explicitly assessed any inequalities along gender and socio-economic axes.

There were also several limitations in our study. We were not able to directly identify associations between neighbourhood features and behaviours. For example, although we found a greater number of public transport stops within some areas, we were unable to assess use of those facilities. We recommend that future studies explore relationships between proximity, accessibility, use, and potential health effects of, local amenities/facilities. We were unable to determine the quality of the infrastructure within 20-minute neighbourhoods, which may vary by area-level deprivation. For example, in Sheffield and New Zealand research showed that although more deprived areas had greater access to greenspace, often this was of poorer quality (Chaudhury *et al.* 2017; Mears *et al.* 2019). Future studies should integrate measures of neighbourhood safety in their research designs.

### 4.4 Conclusions

Children spend a considerable amount of their time within their 20-minute neighbourhood. Overall, attending a school within a 20-minute neighbourhood was associated with spending a greater amount of time closer to home. There was variation by area-level socioeconomic status in what amenities, facilities and environments lie within a 20-minute neighbourhood, highlighting children from the most deprived area have greater proximity to both health benefiting and health harming environments facilities, which collocate within the most deprived areas. We emphasise that the policy should shift beyond a focus solely on access to local facilities and amenities but should instead focus on creating healthy 20-minute neighbourhoods, particularly for the most socioeconomically deprived areas. The pathways between 20-minute neighbourhoods features and children’s health outcomes should also be explored.

## Supplementary Material

Supplementary Material

## Figures and Tables

**Table 1:** Neighbourhood characteristics, justification, and source.

Feature	Specific features	Justification	Access measure	Source:
**School**	Distance from child’s school	The school is a key location where children spend a significant amount of their time (Chambers *et al*. 2017; Jonathan R Olsen *et al*. 2019).	Binary ‘Yes’ if child’s school within 800m of home.	SPACES dataset.
	Primary schools		Count: Number of schools within 800m of home.	OS Points of Interest, March 2015.
**Residential count**	Number of residential locations.	Residential count is included as a factor in the healthy environments index (Whitehead *et al*. 2023).	Count: Number of residential postal addresses within 800m of home.	OS Code Point
**Public transportation stops**	Bus stop	Public transportation provides opportunities to travel and children in more deprived areas have been shown to have greater contact with the transport network. Included as a factor in the healthy environments index (Whitehead *et al*. 2023). The presence or absence of major or minor roads may be associated with healthy environments for children (Whitehead *et al*. 2023).	Count: Number of facilities within 800m of home.	OS Points of Interest, March 2015.
	Railway station:			
	Tram:			
	Underground			
**Road type**	Presence of roads (meters) by road type (major or minor)		Length (km) of road.	OS Open Rods.
**Retail (non-food) Healthy food and drink retail**	Clothing and accessories	Non-food retailers may provide a local destination to spent time. Included as a factor in the healthy environments index (Whitehead *et al*. 2023).	Count: Number of facilities within 800m of home.	OS Points of Interest, March 2015.
	Household, office, leisure and garden Supermarket	Large and medium sized supermarkets provide a large range of healthy and fresh food items at a reasonable and affordable price, compared to smaller convenience stores or ‘corner’ shops.	Count: Number of facilities within 800m of home.	OS Points of Interest, March 2015.
	Medium Supermarket from Convenience store (M&S Simply Food, Morrisons Local, Sainsbury Local, Tesco Metro/Express)			
**Unhealthy food and drink retail**	Fast food and takeaway outlets	Unhealthy and health retailers have been shown to be co-located within similar neighbourhoods (Marek *et al*. 2021).	Count: Number of facilities within 800m of home.	OS Points of Interest, March 2015.
	Fish and chip shops			
	Pubs, bars and inns			
	Alcoholic drinks including off-licences and wholesalers			
**Health**	Primary care (GP)	NHS services are provided free of charge for all persons living in Scotland. The GP is usually the main point of access to medical care. ‘Walk-in-centres’ provide access to urgent medical attention where it is not a life-threatening situation (NHS. 2021).	Count: Number of facilities within 800m of home.	OS Points of Interest, March 2015.
	Walk-in-Centre			
**Places of worship**	Places of worship	Destination has been shown as a location children spend time (Jonathan R Olsen *et al*. 2019).	Count: Number of facilities within 800m of home.	OS Points of Interest, March 2015.
**Libraries**	Library	Destination has been shown as a location children spend time (Jonathan R Olsen *et al*. 2019).	Count: Number of facilities within 800m of home.	OS Points of Interest, March 2015.
**Sports and recreational facilities**	Athletics facilities	The presence of a gymnasium, sports hall, leisure centre and a range of specific sports facilities and pitches were used as indicators of recreational, sports pitches and facilities (Chambers *et al*. 2017; Egli *et al*. 2020; Sharmin and Kamruzzaman 2017; Thomson *et al*. 2003; Villanueva *et al*. 2013).	Count: Number of facilities within 800m of home.	OS Points of Interest, March 2015.
	Golf ranges, courses and clubs.			
	Sports grounds, stadia and pitches			
	Swimming pools			
	Tennis facilities			
	Gymnasiums, sports halls and leisure centres			
**Natural space (including public parks and private gardens)**	Greenspace access point	Playing in green places near home were associated with more time spent in light physical activity and less sedentary behaviour (Lin *et al*. 2022). Green spaces were shown as both frequent and preferred locations children spend time (Egli *et al*. 2020). Greater access to natural space associated with increased time living locally (McCrorie *et al*. 2021).	Count: Number of ‘Public Open Space Access Point’ within 800m of home.	Open Greenspace, July 2017 (Ordnance Survey, 2022).
	Natural space		Proportion: Natural space coverage.	OS MasterMap, 2015.
	Private garden		Proportion: Private Garden coverage.	OS MasterMap, 2015.
	Municipal parks		Proportion: Public parks within 800m of home.	Greenspace Scotland Map
**Land-use mix**	Non-food and retail commercial services	Land-use mix within local area may be an indication of the range of opportunities available (Loebach and Gilliland 2016; Sharmin and Kamruzzaman 2017; Villanueva *et al*. 2016). It is included as a factor in the healthy environments index (Whitehead *et al*. 2023).	Count: Number of facilities within 800m of home.	OS Points of Interest, March 2015.
	Manufacturing and production		Count: Number of facilities within 800m of home.	
	Retail (food and non-retail)		Count: Number of facilities within 800m of home.	
	Public infrastructure, Education and health		Count: Number of facilities within 800m of home.	
	Open Space (‘bodies of water’; landscape features; recreational features.)		Count: Number of ‘bodies of water’; landscape features; recreational features.	
**Urbanicity / Rural definitions**	Datazone 6-fold urban rural classification of residential location	Urbanicity and housing density shown to be associated with children’s time in local neighbourhood (Loebach and Gilliland 2016)	Scottish Government 6-fold urban/rural classification.	2016 Urban Rural Classification (Scottish Government 2018)
**Population density**	Datazone population density at residential location	Urbanicity and housing density shown to be associated with children’s time in local neighbourhood (Loebach and Gilliland 2016).	Population density (per 1,000) of datazone of home location.	2016 mid-year population estimates

**Table 2:** Characteristics of children’s 20-minute neighbourhood by sex, socioeconomic status and urbanicity.

		Size	School		Urban density and transport				Retail				Amenities			Greenspace, sport, and recreation					Land-use diversity		
		Area of 20-minute neighbourhood	School (attends)	School (any)	Residential address count	Public transport	Major road	Minor road	Retail ALL	Retail (non-food)	Healthy food retail	Unhealthy food & drink	Health	Place of worship	Libraries	Sports and recreational facilities	Natural space	Private gardens	Greenspace access point	Municipal parks	Open space	Manufacturing	Public Infrastructure
Data type*		**M2**	**%**	**C**	**C**	**C**	**M**	**M**	**C**	**C**	**C**	**C**	**C**	**C**	**C**	**C**	**A%**	**A%**	**C**	**M2**	**C**	**C**	**C**
Sex	Female	881010	32	1	1017	11	598	9431	4	1	0	1	0	1	0	2	44	27	8	38480	2	2.5	19
	Male	934434	36	1	1160	13	925	10040	4	2	0	1	0	1	0	0	41	27	8	41046	2	3	22
SIMD	1 – Most Deprived	1188297	49	2	2169	28	1272	14804	12	4	1	4	1	2	0	6	37	26	17	81094	3	6	39
	2	1057357	47	1	1619	18	1337	13253	8	2	1	3	1	2	0	4	39	27	12	45568	3	4	32
	3	869690	37	1	1085	10	868	9385	4	1	0	1	0	1	0	0	45	26	8	39647	2	3	21
	4	802168	36	1	649	9	931	7438	3	1	0	0	0	0	0	0	50	23	6	27897	2	2	15
	5 – Least Deprived	886110	23	0	1023	10	512	9483	3	1	0	1	0	0	0	0	41	31	6	36009	2	2	18
Urban/Rural	Large Urban Areas	1106920	33	1	1961	20	1057	14772	8	3	1	3	1	1	0	4	33	31	11	76762	3	4	30
	Other Urban Areas	1024883	33	1	1316	17	1098	11955	4	2	0	2	0	1	0	2	40	28	10	47008	3	3	24
	Accessible Small Towns	780959	32	1	818	10	40	7942	3	1	0	1	0	1	0	0	43	30	7	32475	3	2	19
	Remote Small Towns	774091	64	1	854	13	1415	8432	2.5	1	0	1	0	1	0	1	44	27	14	44002	2.5	3	20
	Accessible Rural Areas	636621	36	0	320	5	157	5090	1	1	0	0	0	0	0	0	63	19	3	11232	1	1	7
	Remote Rural Areas	556161	70	0	57	2	0	3188	1	0	0	0	0	0	0	0	81	12	2	2833	1	2	3
	All	907674	34	1	1095	12	860	9779	4	1	0	1	0	1	0	0	42	27	8	40352	2	3	20

* %: Proportion children with school <800m, C:count of amenity, M:Meter length, M2: meters square, A%: Proportion of area.

**Table 3:** Associations between GPS wear time spent within 20-minute neighbourhoods, sex, socioeconomic status urbanicity and significant neighbourhood features.

	Overall				Weekday				Weekend			
	LL 95%				LL 95%				LL 95%			
	IRR	CI	UL 95% CI	p value	IRR	CI	UL 95%	p value	IRR	CI	UL 95% CI	p value
**Attended school within 800m**												
No	Ref				Ref				Ref			
Yes	1.62	1.5	1.76	<0.001	1.87	1.71	2.05	<0.001	1.12	1.02	1.23	0.02
Sex												
Male	Ref				Ref				Ref			
Female	1.00	0.93	1.07	0.96	1.03	0.95	1.12	0.47	0.96	0.88	1.05	0.34
**Area-level socioeconomic status**												
1 (most deprived)	1.14	1.01	1.28	0.03	1.11	0.96	1.28	0.15	1.19	1.07	1.33	<0.001
2	0.99	0.88	1.11	0.83	0.97	0.83	1.12	0.64	0.98	0.87	1.11	0.76
3	1.09	1.01	1.18	0.03	1.11	1.01	1.22	0.04	1.00	0.88	1.14	1.00
4	0.96	0.87	1.06	0.40	0.95	0.84	1.08	0.45	0.99	0.9	1.09	0.84
5 (least deprived)	Ref				Ref				Ref			
	*(Wald 0.012)*				*(Wald 0.012)*				*(Wald 0.023)*			
**Urban / Rural status**												
Urban	Ref				Ref				Ref			
Rural	0.92	0.85	0.98	0.02	0.90	0.82	0.98	0.02	0.95	0.83	1.09	0.43
**Non-residential diversity**												
	1.05	0.91	1.2	0.53	1.08	0.92	1.25	0.35	0.99	0.83	1.18	0.89
