# Supplementary material for: Inequalities in neighbourhood features within children’s 20-minute neighbourhoods and variation in time spent locally, measured using GPS
Source: Wellbeing Space Soc. Author manuscript; Available in PMC 2023 Dec 9. (PMC7615365; doi:10.1016/j.wss.2023.100174)
Supplement: Supplementary Material [file EMS191886-supplement-Supplementary_Material.pdf]

Supplementary Material

Supplementary Table 1: Characteristics of children's 20-minute neighbourhood by sex, socioeconomic status and urbanicity (800m Euclidean buffer).

		School		Urban density and transport				Retail				Amenities			Greenspace, sport, and recreation					Land-use diversity		
		School (attends)	School (any)	Residential address count	Public transport	Major road	Minor road	Retail ALL	Retail (non-food)	Healthy food retail	Unhealthy food & drink	Health	Place of worship	Libraries	Sports and recreational facilities	Natural space	Private gardens	Greenspace access point	Municipal parks	Open space	Manufacturing	Public Infrastructure
Data type*		**%**	**C**	**C**	**C**	**M**	**M**	**C**	**C**	**C**	**C**	**C**	**C**	**C**	**C**	**A%**	**A%**	**C**	**M2**	**C**	**C**	**C**
Sex	Female	31.8	1	1743	16.9	1502	15877	7	2	0	2	0	1	0	4	56	18	12	77918	4	5	31
	Male	36.2	1	1755	19	1586	17187	8	3	1	2	0	1	0	4	53	18	13	85312	4	6	35
SIMD	1 – Most Deprived	49.1	2	2997	38	1831	21081	18	7	1	7	1	2	1	8	43	21	24	153186	5	10	54
	2	46.9	2	2325	24	1703	19396	12	3	1	4	1	2	0	7	46	20	19	110801	5	9	45
	3	37.1	1	1613	16	1577	14862	6	2	1	3	0	1	0	4	59	17	12	77804	4	5	34
	4	35.6	1	1039	13	1563	12427	5	2	0	1	0	1	0	3	64	15	10	53428	4	4	25
	5 – Least Deprived	23.1	1	1727	17	1460	17651	6	2	0	2	0	1	0	3	54	21	11	85312	4	4	33
Urban/Rural	Large Urban Areas	33.3	2	2928	28	1593	23278	14	6	1	6	1	2	0	8	38	25	18	174447	5	8	49
	Other Urban Areas	33	1	2046	24	1639	18346	9	3	1	3	0	1	0	5	50	20	15	124913	5	6	37
	Accessible Small Towns	31.9	1	1430	16	1439	15318	7	2	1	2	1	1	0	5	61	18	12	65053	5	7	27
	Remote Small Towns	63.6	1	1219	21	1982	13615	7	2	1	3	0	2	0	6	50	17	17	77778	5	8	31
	Accessible Rural Areas	36.2	1	404	6	1177	7851	2	1	0	0	0	0	0	0	83	9	4	23837	3	3	10
	Remote Rural Areas	69.7	0	95	3	956	5365	1	1	0	0	0	0	0	0	88	3	2	8715	2	3	5
	All	34.2	1	1755	19	1551	16883	7	3	1	2	0	1	0	4	54	18	12	81829	4	5	34

*%: Proportion children with school <800m, C:count of amenity, M:Meter length, M2: meters square, A%: Proportion of area.

Supplementary Table 2: Proportion of time spent within 20-minute neighbourhoods by sex, socioeconomic status and urbanicity.

	All			Weekday			Weekend		
	**n**	**Proportion (mean)**	**std. error**	**n**	**Proportion (mean)**	**std. error**	**n**	**Proportion (mean)**	**std. error**
**Gender**									
Female	308	59.9	1.3	308	57.3	1.6	293	67.5	1.4
Male	379	60.1	1.2	379	58.9	1.4	362	64.5	1.3
**Socioeconomic status**									
1 – Most Deprived	57	67.1	3.2	57	66.1	3.8	52	71.8	3.2
2	81	67.1	2.6	81	68.3	3.0	79	63.4	3.1
3	140	62.1	2.1	140	59.8	2.5	132	69.2	2.2
4	180	58.8	1.7	180	57.2	2.0	173	64.7	1.9
5 – Least Deprived	229	55.3	1.4	229	52.5	1.7	219	64.2	1.6
**Urban / Rural**									
Large Urban Areas	213	58.8	1.7	213	57.1	2.0	202	64.6	1.7
Other Urban Areas	203	63.8	1.5	203	62	1.8	195	70	1.7
Accessible Small Towns	69	61.1	2.6	69	60.8	3.1	66	61.7	3.2
Remote Small Towns	22	72.5	5.8	22	73.4	6.3	22	66	6.5
Accessible Rural Areas	116	56.4	2.1	116	53.8	2.5	111	65.3	2.4
Remote Rural Areas	64	52.9	3.0	64	49.7	3.4	59	61.9	3.6
**Total**									
	687	60	0.9	687	65.8	1.1	655	58.2	1.0

Supplementary Table 3: Individual neighbourhood features, adjusted for sex, area-level socioeconomic status and urbanicity (Adjusting for multiple tests of various features, for each outcome).

	Overall				Weekday				Weekend			
**Neighbourhood feature**	IRR	LL 95% CI	UL 95% CI	p value	IRR	LL 95% CI	UL 95% CI	p value	IRR	LL 95% CI	UL 95% CI	p value
Public transport	1.00	1.00	1.00	0.46	1.00	1.00	1.01	0.29	1.00	0.99	1.00	0.82
Retail (non-food)	1.00	1.00	1.00	0.40	1.00	1.00	1.00	0.38	1.00	1.00	1.00	0.95
Healthy food retail	1.02	0.99	1.04	0.14	1.02	0.98	1.06	0.24	1.00	0.97	1.03	0.98
Unhealthy food & drink	0.002	-0.001	0.003	0.06	1.00	1.00	1.00	0.19	1.01	1.00	1.00	0.77
Health	1.01	0.99	1.02	0.22	1.01	0.99	1.03	0.34	1.01	0.99	1.02	0.44
Place of worship	1.02	1.00	1.04	0.04	1.02	1.00	1.05	0.04	1.02	1.00	1.04	0.04
Libraries	1.04	0.96	1.13	0.36	1.06	0.97	1.16	0.21	1.00	0.90	1.10	0.93
Sports and recreational facilities	1.00	1.00	1.01	0.46	1.01	1.00	1.01	0.15	1.00	0.99	1.01	0.99
Natural space	1.00	1.00	1.00	0.29	1.00	1.00	1.00	0.26	1.00	1.00	1.00	0.70
Private gardens	1.00	1.00	1.00	0.99	1.01	1.00	1.01	0.74	1.00	0.99	1.00	0.50
Greenspace access point	1.00	1.00	1.01	0.05	1.01	1.00	1.01	0.05	1.00	1.00	1.01	0.42
Municipal parks	1.00	1.00	1.00	0.13	1.00	0.99	1.00	0.04	1.00	1.00	1.00	0.91
