# Supplementary material for: Inequalities in neighbourhood features within children's 20-minute neighbourhoods and variation in time spent locally, measured using GPS
Source: Wellbeing Space Soc. 2023 Dec;5:100174. doi: 10.1016/j.wss.2023.100174 (PMC7615365; doi:10.1016/j.wss.2023.100174)
Supplement: Supplementary file 1 [file mmc1.docx]

# Supplementary Material

Supplementary Table 1: Characteristics of children’s 20-minute neighbourhood by sex, socioeconomic status and urbanicity (800m Euclidean buffer).

|  |  | School |  | Urban density and transport |  |  |  | Retail |  |  |  | Amenities |  |  | Greenspace, sport, and recreation |  |  |  |  | Land-use diversity |  |  |
| --- | --- | --- | --- | --- | --- | --- | --- | --- | --- | --- | --- | --- | --- | --- | --- | --- | --- | --- | --- | --- | --- | --- |
|  |  | School (attends) | School (any) | Residential address count | Public transport | Major road | Minor road | Retail ALL | Retail (non-food) | Healthy food retail | Unhealthy food & drink | Health | Place of worship | Libraries | Sports and recreational facilities | Natural space | Private gardens | Greenspace access point | Municipal parks | Open space | Manufacturing | Public Infrastructure |
| Data type* |  | **%** | **C** | **C** | **C** | **M** | **M** | **C** | **C** | **C** | **C** | **C** | **C** | **C** | **C** | **A%** | **A%** | **C** | **M2** | **C** | **C** | **C** |
| Sex | Female | 31.8 | 1 | 1743 | 16.9 | 1502 | 15877 | 7 | 2 | 0 | 2 | 0 | 1 | 0 | 4 | 56 | 18 | 12 | 77918 | 4 | 5 | 31 |
|  | Male | 36.2 | 1 | 1755 | 19 | 1586 | 17187 | 8 | 3 | 1 | 2 | 0 | 1 | 0 | 4 | 53 | 18 | 13 | 85312 | 4 | 6 | 35 |
| SIMD | 1 – Most Deprived | 49.1 | 2 | 2997 | 38 | 1831 | 21081 | 18 | 7 | 1 | 7 | 1 | 2 | 1 | 8 | 43 | 21 | 24 | 153186 | 5 | 10 | 54 |
|  | 2 | 46.9 | 2 | 2325 | 24 | 1703 | 19396 | 12 | 3 | 1 | 4 | 1 | 2 | 0 | 7 | 46 | 20 | 19 | 110801 | 5 | 9 | 45 |
|  | 3 | 37.1 | 1 | 1613 | 16 | 1577 | 14862 | 6 | 2 | 1 | 3 | 0 | 1 | 0 | 4 | 59 | 17 | 12 | 77804 | 4 | 5 | 34 |
|  | 4 | 35.6 | 1 | 1039 | 13 | 1563 | 12427 | 5 | 2 | 0 | 1 | 0 | 1 | 0 | 3 | 64 | 15 | 10 | 53428 | 4 | 4 | 25 |
|  | 5 – Least Deprived | 23.1 | 1 | 1727 | 17 | 1460 | 17651 | 6 | 2 | 0 | 2 | 0 | 1 | 0 | 3 | 54 | 21 | 11 | 85312 | 4 | 4 | 33 |
| Urban/Rural | Large Urban Areas | 33.3 | 2 | 2928 | 28 | 1593 | 23278 | 14 | 6 | 1 | 6 | 1 | 2 | 0 | 8 | 38 | 25 | 18 | 174447 | 5 | 8 | 49 |
|  | Other Urban Areas | 33 | 1 | 2046 | 24 | 1639 | 18346 | 9 | 3 | 1 | 3 | 0 | 1 | 0 | 5 | 50 | 20 | 15 | 124913 | 5 | 6 | 37 |
|  | Accessible Small Towns | 31.9 | 1 | 1430 | 16 | 1439 | 15318 | 7 | 2 | 1 | 2 | 1 | 1 | 0 | 5 | 61 | 18 | 12 | 65053 | 5 | 7 | 27 |
|  | Remote Small Towns | 63.6 | 1 | 1219 | 21 | 1982 | 13615 | 7 | 2 | 1 | 3 | 0 | 2 | 0 | 6 | 50 | 17 | 17 | 77778 | 5 | 8 | 31 |
|  | Accessible Rural Areas | 36.2 | 1 | 404 | 6 | 1177 | 7851 | 2 | 1 | 0 | 0 | 0 | 0 | 0 | 0 | 83 | 9 | 4 | 23837 | 3 | 3 | 10 |
|  | Remote Rural Areas | 69.7 | 0 | 95 | 3 | 956 | 5365 | 1 | 1 | 0 | 0 | 0 | 0 | 0 | 0 | 88 | 3 | 2 | 8715 | 2 | 3 | 5 |
|  | All | 34.2 | 1 | 1755 | 19 | 1551 | 16883 | 7 | 3 | 1 | 2 | 0 | 1 | 0 | 4 | 54 | 18 | 12 | 81829 | 4 | 5 | 34 |

*%: Proportion children with school <800m, C:count of amenity, M:Meter length, M2: meters square, A%: Proportion of area.

Supplementary Table 2: Proportion of time spent within 20-minute neighbourhoods by sex, socioeconomic status and urbanicity.

|  | **All** |  |  | **Weekday** |  |  | **Weekend** |  |  |
| --- | --- | --- | --- | --- | --- | --- | --- | --- | --- |
|  | **n** | **Proportion (mean)** | **std. error** | **n** | **Proportion (mean)** | **std. error** | **n** | **Proportion (mean)** | **std. error** |
| **Gender** |  |  |  |  |  |  |  |  |  |
| Female | 308 | 59.9 | 1.3 | 308 | 57.3 | 1.6 | 293 | 67.5 | 1.4 |
| Male | 379 | 60.1 | 1.2 | 379 | 58.9 | 1.4 | 362 | 64.5 | 1.3 |
| **Socioeconomic status** |  |  |  |  |  |  |  |  |  |
| 1 – Most Deprived | 57 | 67.1 | 3.2 | 57 | 66.1 | 3.8 | 52 | 71.8 | 3.2 |
| 2 | 81 | 67.1 | 2.6 | 81 | 68.3 | 3.0 | 79 | 63.4 | 3.1 |
| 3 | 140 | 62.1 | 2.1 | 140 | 59.8 | 2.5 | 132 | 69.2 | 2.2 |
| 4 | 180 | 58.8 | 1.7 | 180 | 57.2 | 2.0 | 173 | 64.7 | 1.9 |
| 5 – Least Deprived | 229 | 55.3 | 1.4 | 229 | 52.5 | 1.7 | 219 | 64.2 | 1.6 |
| **Urban / Rural** |  |  |  |  |  |  |  |  |  |
| Large Urban Areas | 213 | 58.8 | 1.7 | 213 | 57.1 | 2.0 | 202 | 64.6 | 1.7 |
| Other Urban Areas | 203 | 63.8 | 1.5 | 203 | 62 | 1.8 | 195 | 70 | 1.7 |
| Accessible Small Towns | 69 | 61.1 | 2.6 | 69 | 60.8 | 3.1 | 66 | 61.7 | 3.2 |
| Remote Small Towns | 22 | 72.5 | 5.8 | 22 | 73.4 | 6.3 | 22 | 66 | 6.5 |
| Accessible Rural Areas | 116 | 56.4 | 2.1 | 116 | 53.8 | 2.5 | 111 | 65.3 | 2.4 |
| Remote Rural Areas | 64 | 52.9 | 3.0 | 64 | 49.7 | 3.4 | 59 | 61.9 | 3.6 |
| **Total** |  |  |  |  |  |  |  |  |  |
|  | 687 | 60 | 0.9 | 687 | 65.8 | 1.1 | 655 | 58.2 | 1.0 |

Supplementary Table 3: Individual neighbourhood features, adjusted for sex, area-level socioeconomic status and urbanicity (Adjusting for multiple tests of various features, for each outcome).

| **Neighbourhood feature** | Overall |  |  |  | Weekday |  |  |  | Weekend |  |  |  |
| --- | --- | --- | --- | --- | --- | --- | --- | --- | --- | --- | --- | --- |
|  | IRR | LL 95% CI | UL 95% CI | p value | IRR | LL 95% CI | UL 95% CI | p value | IRR | LL 95% CI | UL 95% CI | p value |
| Public transport | 1.00 | 1.00 | 1.00 | 0.46 | 1.00 | 1.00 | 1.01 | 0.29 | 1.00 | 0.99 | 1.00 | 0.82 |
| Retail (non-food) | 1.00 | 1.00 | 1.00 | 0.40 | 1.00 | 1.00 | 1.00 | 0.38 | 1.00 | 1.00 | 1.00 | 0.95 |
| Healthy food retail | 1.02 | 0.99 | 1.04 | 0.14 | 1.02 | 0.98 | 1.06 | 0.24 | 1.00 | 0.97 | 1.03 | 0.98 |
| Unhealthy food & drink | 0.002 | -0.001 | 0.003 | 0.06 | 1.00 | 1.00 | 1.00 | 0.19 | 1.01 | 1.00 | 1.00 | 0.77 |
| Health | 1.01 | 0.99 | 1.02 | 0.22 | 1.01 | 0.99 | 1.03 | 0.34 | 1.01 | 0.99 | 1.02 | 0.44 |
| Place of worship | 1.02 | 1.00 | 1.04 | 0.04 | 1.02 | 1.00 | 1.05 | 0.04 | 1.02 | 1.00 | 1.04 | 0.04 |
| Libraries | 1.04 | 0.96 | 1.13 | 0.36 | 1.06 | 0.97 | 1.16 | 0.21 | 1.00 | 0.90 | 1.10 | 0.93 |
| Sports and recreational facilities | 1.00 | 1.00 | 1.01 | 0.46 | 1.01 | 1.00 | 1.01 | 0.15 | 1.00 | 0.99 | 1.01 | 0.99 |
| Natural space | 1.00 | 1.00 | 1.00 | 0.29 | 1.00 | 1.00 | 1.00 | 0.26 | 1.00 | 1.00 | 1.00 | 0.70 |
| Private gardens | 1.00 | 1.00 | 1.00 | 0.99 | 1.01 | 1.00 | 1.01 | 0.74 | 1.00 | 0.99 | 1.00 | 0.50 |
| Greenspace access point | 1.00 | 1.00 | 1.01 | 0.05 | 1.01 | 1.00 | 1.01 | 0.05 | 1.00 | 1.00 | 1.01 | 0.42 |
| Municipal parks | 1.00 | 1.00 | 1.00 | 0.13 | 1.00 | 0.99 | 1.00 | 0.04 | 1.00 | 1.00 | 1.00 | 0.91 |
